# Donation Physician Specialists and Missed Organ Donation Opportunities

**DOI:** 10.1001/jamanetworkopen.2025.26067

**Published:** 2025-08-07

**Authors:** Andreas Kramer, Kerry Holliday, Rachel Wilkins, Selena Au, Philippe Couillard, Christopher Doig, Julie Kromm, Andrea Soo, Dennis Djogovic

**Affiliations:** 1 Give Life Alberta, Edmonton, Alberta; 2 Department of Critical Care Medicine, University of Calgary, Calgary, Alberta, Canada; 3 Department of Critical Care Medicine, University of Alberta, Edmonton, Alberta, Canada

## Abstract

**Importance:**

Donation physicians (DPs) are usually critical care specialists whose responsibilities may include assessment of donation eligibility, care of potential donors, death determination, education, and advocacy. While DP programs have been implemented in some countries with the aim of optimizing the organ donation process, there are few studies evaluating their effectiveness.

**Objective:**

To assess the association of a DP program with key performance metrics.

**Design, Setting, and Participants:**

This cohort study used interrupted time series analysis to compare baseline data from intensive care units and emergency departments in a Canadian province (December 2019 to February 2020 and March 2021 to May 2021) with data following the initial 3 years of a DP program (July 2021 to June 2024). Donation data included consecutive deceased critically ill patients with various forms of brain injury who received mechanical ventilation in the last 12 hours of life.

**Exposure:**

Implementation of a province-wide DP program coupled with regular audit and feedback aimed at identifying and minimizing missed donation opportunities.

**Main Outcomes and Measures:**

Missed organ donation opportunities, defined as occurring when families of eligible potential donors were not given the option of donation. Secondary outcomes included appropriate notification of the provincial donation organization, donors per million population, and median number of organs transplanted per donor.

**Results:**

Over 42 months, there were 1072 eligible potential donors, including 635 (59%) following death by neurologic criteria and 437 (41%) following death by circulatory criteria. The median (IQR) age was 44.6 (30.8-58.3) years, 662 (62%) were male, and the most common cause of death was hypoxic-ischemic brain injury. During the initial 36 months of the DP program, 129 of 942 (14%) eligible potential donors were missed, compared with 43 of 123 (33%) during 6 months of baseline data (*P* < .001). The monthly proportion of missed cases decreased by 10.9% (95% CI, −22.0% to 0.3%; *P* = .06) immediately following the start of the program and then declined −0.7% (95% CI, −0.9% to −0.5%; *P* < .001) per month. Appropriate notification of the provincial organ donation organization increased 0.9% (95% CI, 0.6% to 1.3%, *P* < .001) per month. The annual donation rate increased from 14.0 to 23.7 donors per million. The median (IQR) number of organs transplanted per donor did not change (4 [3-5]; *P* = .69). Missed opportunities occurred in 3% of eligible potential donors when the most responsible physician was a DP and 17% when it was not (*P* < .001).

**Conclusions:**

In this cohort study of consecutive eligible potential organ donors, implementation of a novel DP program was associated with a sustained reduction in missed organ donation opportunities, increased referrals, and a higher deceased donation rate.

## Key Points

**Question:**

Are donation physicians associated with key donation performance metrics within a health care system?

**Findings:**

In this cohort study including 1072 eligible potential donors, a novel donation physician program was associated with a significant and sustained reduction in missed organ donation opportunities, increased referrals to an organ donation organization, and a higher overall donation rate.

**Meaning:**

These results suggest that donation physician programs may improve donation performance within a health care system.

## Teaser

This cohort study of Canadian doctors examines the association of a donation physician program to facilitate organ donation with reductions in missed organ donation opportunities.

## Introduction

Transplantation is optimal treatment for many patients with end-stage organ failure, and deceased donation is the primary source of organs. Most donors are critically ill patients with various forms of brain injury that progress to death by neurologic criteria (DNC) or undergo withdrawal of life-sustaining measures (WLSM) because of a poor prognosis, with organs recovered following death by circulatory criteria (DCC).^1^

Numerous interventions have been proposed to optimize donation systems. One strategy adopted in several countries, including Spain, the UK, Australia, and most Canadian provinces, is implementation of a donation physician (DP) role with enhanced training and expertise in deceased organ donation processes. Responsibilities of DPs may include supportive roles in assessment of donation eligibility, communication with families, care of potential donors, guideline-directed death determination, provision of professional education, quality improvement, administration, and donation advocacy.^2,3,4,5,6,7^ In the US, the role of DPs has been limited, although success has been reported with an intensivist-led donor management program.^4^

Given the growing complexities of deceased donation, there is a strong rationale for special expertise in this area, but there are few studies evaluating the impact of DP programs. Previous research found implementation of hospital-based “transplantation coordinators,” some of whom were physicians, to be associated with higher consent, conversion, organ donation, and transplantation rates.^8,9,10,11,12,13,14,15,16^ Limitations of these studies include that they were disproportionately performed in a small number of countries, did not provide baseline data prior to availability of DPs, involved multiple concomitant interventions, and did not conduct inferential statistical analyses.^8^ Our objective was to prospectively evaluate system-wide implementation of a DP program in a Canadian province with a population of about 5 million.

## Methods

This was a quality improvement project that underwent ethics review through the Alberta Research Ethics Community Initiative (ARECCI).^17^ This study followed the Strengthening the Reporting of Observational Studies in Epidemiology (STROBE) reporting guideline.

### SEND Physician Program

In 2021, to be consistent with national recommendations, the Government of Alberta funded a DP program.^18^ The goal was to promote excellence in care across the organ donation continuum, beginning with evidence-based assessment of prognosis and progressing to routine consideration of donation for patients approaching end of life, communication with the provincial organ donation organization (ODO), donor management, and death determination.

To more comprehensively describe the services provided, including for patients that do not necessarily go on to become potential organ donors, the term *specialist in end-of-life care, neuroprognostication, and donation* (SEND) was coined. Professional development occurs through regular educational rounds, required reading, conference and course attendance, and case reviews. SEND physicians are available across the province, on a rotating basis, for consultation from critical care and emergency medicine (EM) professionals and donation coordinators. In Alberta, donation after DCC proceeds only after 2 expert physicians have provided written opinions concluding that the prognosis is poor. SEND physicians commonly provide this service or act as the second physician in death determination prior to organ recovery.^19^

The SEND program emphasizes evidence-based neuroprognostication, avoiding premature decisions about WLSM, and strict separation between goals of care considerations and later discussions about organ donation.^20,21^ Education regularly focuses on contemporary neuroprognostication research and guidelines. Several SEND physicians have formal neurocritical care training and contribute significantly to education. SEND physicians comply with Canadian ethics guidance for DPs.^22^ Performance of the program is not evaluated based on the overall number of organ donors or achieving a particular consent rate. Rather, key performance indicators are system-wide avoidance of missed donation opportunities and appropriate notification of the provincial ODO.

There are 27 SEND physicians that represent all 16 hospitals across the province with intensive care units (ICUs), including academic and community hospitals and major urban and smaller regional centers. Most SEND physicians are adult intensivists, but there is also representation from pediatric critical care, emergency medicine, and cardiology. SEND physicians are remunerated with a fixed monthly stipend that is not based on any performance metrics. Previously, 2 physicians provided limited clinical and administrative support for the entire province.

### Medical Record Reviews

Collectively, SEND physicians systematically audit deaths in critically ill patients across Alberta for missed organ donation opportunities. Regular audits did not occur prior to the SEND program. Cases are reviewed if the cause of death is brain injury (hypoxic-ischemic, traumatic, cerebrovascular, or other) and the patient had been mechanically ventilated in the last 12 hours of life. Reviews are limited to brain-injured patients because these account for more than 90% of deceased donors in Canada and relatively few patients that die from other causes are eligible for donation after DCC.^23,24^

Patients are defined as eligible potential donors if they meet each of the following criteria: (1) documented as having progressed to DNC, suspected to have progressed to DNC based on retrospective review, or developed DCC within 2 hours of WLSM; (2) no overt contraindication to organ donation; (3) 1 or more transplantable organ (kidney[s], liver, lung[s], or heart); and (4) sufficient physiologic stability for organ allocation (arterial oxygen saturation levels of 90% or higher with FiO_2_ 100% or lower and stable mean arterial pressure of 60 mm Hg or higher [to convert to kilopascals, multiply by 0.133] with norepinephrine 0.5 µg/kg/min or lower). Disagreements regarding contraindications for donation, viability of organs, or physiologic stability are adjudicated by medical directors of the ODO. For donation after DNC, there is no age threshold. For donation after DCC, the current threshold in Alberta is age 70 years.

A missed donation opportunity is defined as occurring when there is no evidence in the medical record that the alternate decision maker of an eligible potential donor was approached regarding organ donation. Exceptions are made when no decision maker can be found, the medical examiner does not authorize donation, or patients’ families are unwilling to accept the diagnosis of DNC. Definitions for medical record review are consistent with those of national recommendations, modified to reflect current eligibility criteria of Alberta transplantation programs.^25^ Although some international programs utilize organs from donors with DCC up to 3 hours after WLSM, this is currently not common practice in Canada.^21,23,24,25,26^

Medical record reviews are conducted within 3 months after deaths. Reviews are initially conducted both by a SEND physician and administrative staff. Potential missed cases are reviewed by a second SEND physician, with disagreements resolved by discussion and, if necessary, a third reviewer. SEND physicians do not review cases in which they were personally involved. This methodology has previously been shown to have good interobserver agreement.^27^ Cases can also be identified as potentially missed by administrative staff, even if initially categorized by a SEND physician as “not missed,” and referred for second opinion.

Reporting tools were developed to share missed opportunities with front-line health care professionals, critical care and emergency medicine leaders, and hospital administrators, with the intention of promoting accountability and avoiding future cases. Feedback reports were developed in partnership with the Alberta Physician Learning Program and are provided quarterly, accompanied by narrative descriptions of cases that were missed with suggestions for quality improvement (eFigure 1 in Supplement 1).

### Outcomes 

The primary outcome was the proportion of eligible potential organ donors that were missed. Secondary outcomes included the proportion of eligible potential donors referred to the ODO and the overall number of deceased organ donors in Alberta. We also recorded the proportion of eligible potential donors that became actual donors and number of organs transplanted per donor.

We compared outcomes before and for 3 years following implementation of the SEND program (July 1, 2021). We collected 6 months of baseline data, including 3 months immediately prior to the start of the program and, to attenuate effects of the COVID-19 pandemic, December 1, 2019, to February 28, 2020. Alberta legislation was modified on April 1, 2023, to mandate referral of patients at end of life to the provincial ODO. We therefore created additional interrupted time series models where change in legislation was considered as an intervention. No other systematic changes occurred in Alberta during the timeframe of interest. Finally, we assessed outcomes based on whether a SEND physician was the MRP at the time of death.

### Statistical Analysis

Continuous data were reported as medians with interquartile ranges and compared using Kruskal-Wallis tests. Categorical data were described as proportions and compared using χ^2^ analysis. Interrupted time series analysis was used to assess associations between initiation of the SEND program and outcomes of interest. We used segmented linear regression with terms for time (months), implementation (before and after), and an interaction term between time and implementation. We did not find strong evidence of autocorrelation and seasonality, so did not incorporate these variables in the models. Multivariable logistic regression was used to examine associations between physician characteristics (DP or non-DP) and donation metrics. Analyses were performed in SAS Enterprise version 8.3 (SAS Institute) and R version 4.3.0 (R Project for Statistical Computing). Two-sided *P* < .05 were considered statistically significant.

## Results

**Figure 1.**
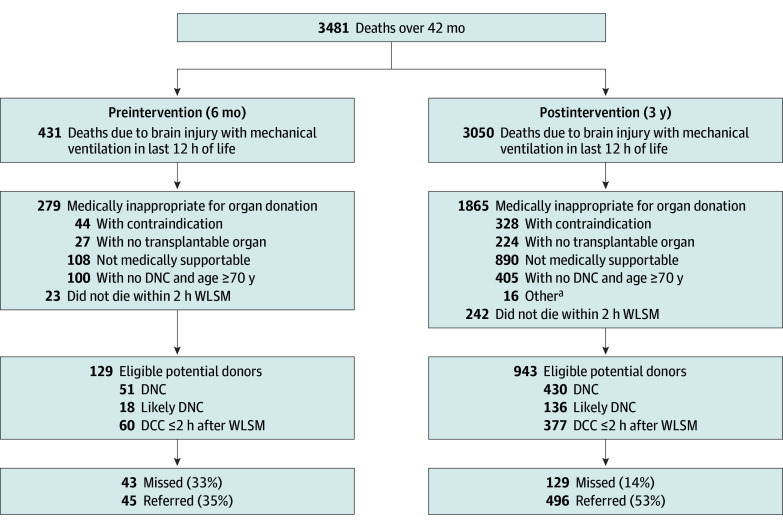
Categorization of Deaths in Province-Wide Medical Record Review Comparison of deaths before and after initiation of donation physician program July 1, 2021. DCC indicates death by circulatory criteria; DNC, death by neurologic criteria; SEND, specialist in end-of-life care, neuro-prognostication, and donation; WLSM, withdrawal of life-sustaining measures ^a^Other reasons included family unaccepting of death or poor prognosis (3 deaths), no next-of-kin (2 deaths), not a neurologic cause of death (4 deaths), no longer ventilated at time of transition to comfort care (2 deaths), rapid WLSM because initiation was contrary to stated goals of care (4 deaths), and family requested end-of-life care without terminal extubation (1 death).

Over 42 months, there were 3481 deaths meeting inclusion criteria, with a median (IQR) age of 58.7 (38.8-70.8) years; 2161 participants 62% were male, and 1871 (54%) died of hypoxic ischemic brain injury (Figure 1). Following initiation of the DP program, there were 3050 deaths, of which 1865 (61%) were categorized as medically inappropriate for organ donation because patients had overt contraindications, had no transplantable organs, were not medically supportable, exceeded age criteria, or other factors. Another 242 were eligible for donation after DCC, but did not die within 2 hours of WLSM. Of 943 eligible potential donors (median [IQR] age, 44 [31-58] years; 573 male [61%]), there were 430 that were formally diagnosed with DNC, 136 that were suspected of DNC (never confirmed), and 377 with DCC within 2 hours of WLSM. During the 6 months of baseline data collection, there were 129 eligible potential donors.

There were no significant differences in characteristics of eligible potential donors before and after implementation of the DP program (Table 1). There were also no differences in the preintervention period based on whether it was prior to or during the COVID-19 pandemic (eTable in Supplement 1).

**Table 1.** Characteristics of Eligible Potential Organ Donors Prior to and Following Initiation of the Provincial Donation Physician Program

Characteristics	Potential Donors, No. (%)		*P* value
	Pre-SEND Program (n = 129)	Post-SEND Program (n = 943)	
Age IQR, y	51 (32-61)	44 (31-58)	.16
Sex			
Male	89 (69)	573 (61)	.07
Female	40 (31)	370 (39)	
Cause of death			
HIBI	73 (57)	559 (59)	.83
Cerebrovascular	30 (23)	214 (23)	
TBI	16 (12)	112 (12)	
Other	10 (8)	58 (6)	
Location of death			
ICU	125 (97)	883 (94)	.14
ED	4 (3)	60 (6)	
Zone			
Edmonton	54 (42)	427 (45)	.75
Calgary	60 (47)	417 (44)	
Regional	15 (12)	99 (11)	
Eligible potential donor type			
DNC	69 (53)	566 (60)	.16
DCC	60 (47)	377 (40)	

Abbreviations: DCC, death by circulatory criteria; DNC, death by neurologic criteria; ED, emergency department; HIBI, hypoxic ischemic brain injury; ICU, intensive care unit; SEND, specialist in end-of-life care, neuroprognostication, and donation; TBI, traumatic brain injury.

The proportion of missed organ donation opportunities in the 6 months prior to the start of the SEND program was 43 of 129 (33%) compared with 129 of 943 (14%) over the subsequent 3 years (Figure 1) (*P* < .001). Interobserver agreement in adjudicating missed opportunities was excellent (κ, 0.91; 95% CI, 0.88-0.94). The corresponding proportion of patients referred to the ODO was 45 of 129 (35%) and 496 of 943 (53%), respectively (*P* < .001). This finding was similar with inclusion of patients that did not die within 2 hours of WLSM.

**Figure 2.**
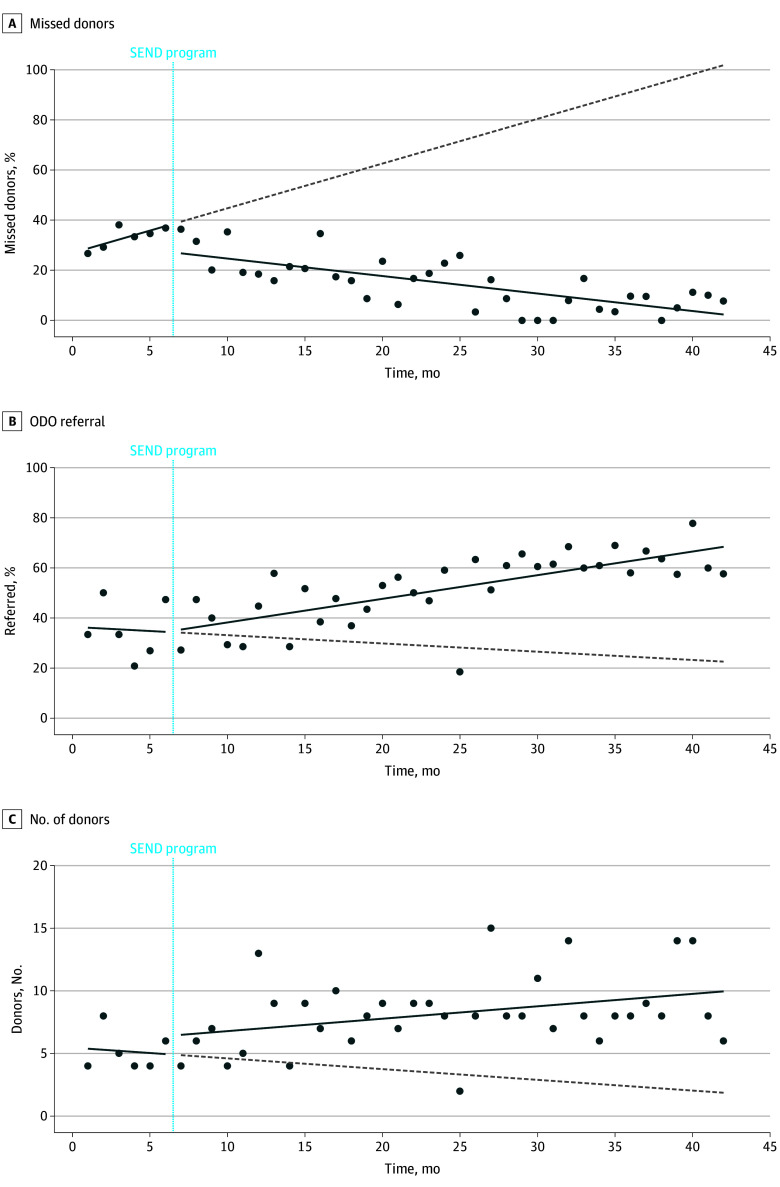
Donation Metrics Over Time Following Implementation of a Provincial Donation Physician Program The Alberta donation physician program commenced July 1, 2021. The dashed gray line represents the counterfactual, namely the expected results based on trend prior to implementation of the donation physician program. ODO indicates organ donation organization; SEND, specialist in end-of-life care, neuro-prognostication, and donation.

In an interrupted time series model, there was no significant change over time in missed opportunities during the 6 months of baseline data collection (1.8% per month; 95% CI, −1.6% to 5.1% per month; *P* = .29). Following the start of the SEND program, there was an initial drop of 10.9% (95% CI, −22.0% to 0.3%) (*P* = .06), with subsequent decline over the ensuing 36 months at a rate of −0.7% (95% CI, −0.9% to −0.5% per month; *P* < .001) (Figure 2). The rate of referral to the provincial organ donation program was stagnant during the 6 months of baseline data collection (−0.3% [95% CI, −5.2% to 4.5%] per month; *P* = .89), but increased at a rate of 0.9% (95% CI, 0.6% to 1.3%) per month (*P* < .001) following the start of the SEND program (Figure 2).

The deceased donation rate in Alberta increased following the start of the SEND program (0.10 [95% CI, 0.01-0.19] per month; *P* = .03), from 14.0 donors per million per year during the baseline 6 months to 23.7 donors per million in the last 6 months of the program (Figure 2). The median (IQR) number of organs recovered and transplanted per donor, with bilateral lung transplantation considered 1 organ, was 4 (3-6) prior to and 4 (3-5) following the start of the SEND program (*P* = .48).

Following the change in Alberta legislation beginning April 1, 2023, there was an initial drop in missed opportunities (−9.0% [95% CI, −17.4% to −0.5%]; *P* = .04), but a subsequent increment (1.1% [95% CI, 0.2% to 2.0%] per month; *P* = .02) (eFigure 2 in Supplement 1). There was an initial increment in the proportion of patients referred that was not statistically significant (11.3% [95% CI, −1.7% to 24.3%] per month; *P* = .09) and no subsequent change over time (0% [95% CI, −1.2% to 1.2%] per month; *P* = .95) (eFigure 3 in Supplement 1). There was no statistically significant change in the number of organ donors either immediately (−0.4% [95% CI, −4.1% to 3.3%]; *P* = .82) or over time (0.1% [95% CI, −0.3% to 0.4%] per month; *P* = .75) (eFigure 4 in Supplement 1).

SEND physicians were the MRP at the time of death in 232 of 943 (25%) eligible potential donors. Missed donation opportunities occurred in 3% (8 of 232 opportunities) when the MRP was a SEND physician, compared with 17% (121 of 711 opportunities) when it was not (*P* < .001) (Table 2). The proportion of missed opportunities declined regardless of whether the MRP was (23% before and 3% after; *P* < .001) or was not (38% before and 17% after; *P* < .001) a SEND physician. Referrals to the ODO were more common when the MRP was a SEND physician (65% vs 49%; *P* < .001), especially for potential DCC donors (54% vs 28%; *P* < .001) (Table 2).

**Table 2.** Comparison of Outcomes Among Eligible Potential Donors Based on Whether the Most Responsible Physician at the Time of Death Was Part of the SEND Program

Characteristics	Potential donations, No./total No. (%)		*P* value
	SEND (n = 232)	Not SEND (n = 711)	
Missed organ donation opportunity			
Overall	8 (3)	121 (17)	<.001
DNC	1/148 (1)	28/418 (7)	.004
DCC	7/84 (8)	93/293 (32)	<.001
Referral to ODO			
Overall	151 (65)	345 (49)	<.001
DNC	106/148 (72)	264/418 (63)	.06
DCC	45/84 (54)	82/293 (28)	<.001
Consent rate			
Overall	98/217 (45)	226/557 (41)	.25
DNC	76/145 (52)	189/375 (50)	.68
DCC	22/72 (31)	37/182 (20)	.08
Proportion utilized donors			
Overall	91 (39)	205 (29)	.003
DNC	73/148 (49)	179/418 (43)	.17
DCC	18/84 (21)	26/293 (9)	.002
Organs transplanted per donor, No. (IQR)^a^	4 (3-5)	4 (3-5)	.69

Abbreviations: DCC, death by circulatory criteria; DNC, death by neurologic criteria; ODO, organ donation organization; SEND, Specialist in End-of-Life Care, Neuro-prognostication and Donation.

^a^ Double lung transplantation considered one organ.

In multivariable analysis adjusting for age, sex, cause of death, unit (EM or ICU), hospital location (urban or regional), and type of eligible potential donor (DNC or DCC), there were fewer missed opportunities when the MRP was a SEND physician (OR, 0.20 [95% CI, 0.09-0.43]; *P* < .001) and more ODO referrals (OR, 2.00 [95% CI, 1.45-2.85]; *P* < .001). The proportion of eligible potential donors from whom at least 1 organ was transplanted was significantly higher for SEND physicians, but only with DCC (21% vs 9%; *P* = .002). Consent rate and organs recovered and transplanted per donor did not differ.

## Discussion

Implementation of a DP program in Alberta was associated with a sustained reduction in missed organ donation opportunities, increased referral of eligible potential donors, and a higher donation rate. Although there have been other DP programs implemented around the world, our study is unique in that the impact was prospectively assessed.

At baseline, a third of eligible potential donors were missed. Following the start of the DP program, there was a relatively rapid drop in missed opportunities that continued over the course of the next 36 months, reaching 7% in the last 6 months of the study. This improvement occurred even though characteristics of eligible potential donors did not change.

The Alberta DP program is multifaceted and improvements in donation metrics could be attributable to several factors. DP representation in ICUs across the province improves donation awareness and increases education for critical care professionals. DPs regularly participate in medical record reviews of missed opportunities and in providing feedback to front-line professionals at their hospitals. Finally, DPs were the MRP at the time of death in a quarter of eligible potential donors, and their involvement was associated with improved donation metrics.

Audits of donation performance are recommended to identify quality improvement opportunities.^28^ In our DP program, medical record reviews are performed by physicians who are experts in the care of critically ill patients and understand potential barriers to donation at their local hospitals. A previous audit of donation activity in Alberta found that when reviews were performed by nonphysicians or utilizing administrative data, donation potential was overestimated.^27^ It is likely that feedback provided by physician members of local teams has a high degree of credibility and, in turn, potential to influence change.

Missed organ donation opportunities are preventable errors that may result in patients not receiving much-needed transplants and should therefore be regarded as “never events.”^29^ Failure to identify donation potential and offer the option of donation also deprives patients and families of an opportunity for altruism. Families of organ donors commonly describe the act of giving as providing a sense of closure and purpose in an otherwise tragic situation, which helps them in their grieving.^30,31^ For these reasons, missed donation opportunities are the key performance indicator for our DP program and were the primary outcome for this study. There are sometimes legitimate reasons why organ donation is not offered, such as medical examiner refusal or lack of family acceptance of DNC. However, whenever appropriate, donation should be offered as part of end-of-life care.

The absolute number of deceased organ donors is a nuanced metric that is influenced by multiple factors, some of which are not necessarily related to system performance, which is why it was considered a secondary outcome.^32,33^ Even so, the donation rate in Alberta increased following the start of the DP program by almost 70%. Donation rates are high in some countries that do not have DPs, such as the US, and it is likely that DP implementation would have variable impact across regions. The cost investment for this program was approximately $700 000 (CAD) per year. Although we did not perform a cost-effectiveness analysis, considering that kidney transplantation is both more efficacious and less costly than other forms of renal replacement therapy, it is likely that our DP program was highly cost-effective.^34^

Routine referral to an ODO is required by legislation in many jurisdictions, including Alberta. Mandatory consideration of donation potential, followed by offering donation when eligible, has been legally required in Alberta since 2009. Legislation changed in 2023 to require notification of the ODO irrespective of eligibility.^35^ Although ICU and EM staff were notified, with regular reminders, of new legislation, we found this change to be associated with only modest, statistically nonsignificant improvement in missed opportunities and ODO referral rate.

The number of organs recovered per donor did not change with DP implementation. We did not specifically audit compliance with national evidence-based recommendations for care of DNC donors.^36^ However, these recommendations are directly incorporated into a province-wide electronic order set that is used in every donor. Thus, management of deceased organ donors is highly standardized, with consistent physiologic goals, mechanical ventilation settings, and use of donor-specific treatments.^35^ It was therefore not expected that there would be major changes in donor management with a DP program.

Previous studies have not assessed whether donation metrics differ depending on characteristics of the MRP. We found that when the MRP was a DP, there were fewer missed opportunities and more ODO referrals. Although this affected provincial metrics, our observation that outcomes improved over time regardless of whether the MRP was a DP suggests that the program was associated with other positive outcomes for system performance.

Studies evaluating DP programs have been summarized in a systematic review.^8^ Most assessed donation metrics following implementation of multi-disciplinary donation and transplantation coordination teams, rather than specifically evaluating DP programs in a health care system that already had donation coordinators. In several cases, there were other system changes that occurred concomitantly with DP programs, making causal inferences unclear. Evaluated outcomes were primarily annual donors per million population, consent and conversion rates, rather than missed donation opportunities. None of the studies utilized conventional methodology for interrupted time series analysis.^37^

### Strengths and Limitations

Strengths of our study were its prospective, population-based design and detailed data from consecutive deaths in critically ill patients. Missed organ donation opportunities is a novel and important performance indicator that health care systems should strive to eliminate.

A limitation of this study is that patients with conditions other than brain injuries can sometimes be organ donors after DCC. However, nonneurologic deaths account for fewer than 10% of deceased donors in Canada.^23,24^ Assessing all deaths in critically ill patients would have greatly increased the number of medical records that required review, while yielding few additional missed donation opportunities. The deceased organ donation rate in Alberta was relatively low at the start of the DP program, with a high rate of missed donation opportunities, particularly following DCC. Implementation of comparable DP programs in jurisdictions with higher donation rates may not be associated with the same degree of improvement. It remains possible that there may have been unrecognized factors, apart from the DP program, that contributed to improved metrics. There is some potential for misclassification in retrospective assessment of missed donation opportunities; however, all records were reviewed in duplicate and interobserver agreement was excellent.

## Conclusions

In this cohort study of consecutive patients with devastating brain injury, we found that system-wide implementation of a DP program with a mandate to optimize donation-related activities, coupled with regular medical record reviews and feedback to frontline health care professionals regarding donation performance, was associated with a significant and sustained reduction in missed organ donation opportunities, increased referral to the provincial ODO, and a higher number of deceased organ donors.

## Notes

Supplement 1. **eFigure 1.** Example of a Quarterly Report Sent to ICU Medical Directors **eTable.** Characteristics of Eligible Potential Organ Donors Prior to Initiation of a Donation Physician Program Based on Whether Death Occurred Prior to, or During the COVID-19 Pandemic **eFigure 2.** Interrupted Time Series Analysis Comparing Missed Organ Donation Rate per Month Before and After Mandatory Referral Legislation in Alberta **eFigure 3.** Interrupted Time Series Analysis Comparing Referral of Eligible Potential Organ Donors, by Month, Before and After Mandatory Referral Legislation in Alberta **eFigure 4.** Interrupted Time Series Analysis Comparing the Number of Deceased Organ Donors per Month Before and After Mandatory Referral Legislation in Alberta

Supplement 2.
Data Sharing Statement
