# Supplementary material for: Donation Physician Specialists and Missed Organ Donation Opportunities
Source: JAMA Netw Open. 2025 Aug 7;8(8):e2526067. doi: 10.1001/jamanetworkopen.2025.26067 (PMC12332632; doi:10.1001/jamanetworkopen.2025.26067)
Supplement: Supplement 1. — eFigure 1. Example of a Quarterly Report Sent to ICU Medical Directors eTable. Characteristics of Eligible Potential Organ Donors Prior to Initiation of a Donation Physician Program Based on Whether Death Occurred Prior to, or During the COVID-19 Pandemic eFigure 2. Interrupted Time Series Analysis Comparing Missed Organ Donation Rate per Month Before and After Mandatory Referral Legislation in Alberta eFigure 3. Interrupted Time Series Analysis Comparing Referral of Eligible Potential Organ Donors, by Month, Before and After Mandatory Referral Legislation in Alberta eFigure 4. Interrupted Time Series Analysis Comparing the Number of Deceased Organ Donors per Month Before and After Mandatory Referral Legislation in Alberta [file jamanetwopen-e2526067-s001.pdf]

Supplemental Online Content

Kramer A, Holliday K, Wilkins R, et al. Donation physicians on missed organ donation opportunities. *JAMA Netw Open.* 2025;8(8):e2526067. doi:10.1001/jamanetworkopen.2025.26067

eFigure 1. Example of a Quarterly Report Sent to ICU Medical Directors

eTable. Characteristics of Eligible Potential Organ Donors Prior to Initiation of a Donation Physician Program Based on Whether Death Occurred Prior to, or During the COVID-19 Pandemic

eFigure 2. Interrupted Time Series Analysis Comparing Missed Organ Donation Rate per Month Before and After Mandatory Referral Legislation in Alberta

eFigure 3. Interrupted Time Series Analysis Comparing Referral of Eligible Potential Organ Donors, by Month, Before and After Mandatory Referral Legislation in Alberta

eFigure 4. Interrupted Time Series Analysis Comparing the Number of Deceased Organ Donors per Month Before and After Mandatory Referral Legislation in Alberta

This supplemental material has been provided by the authors to give readers additional information about their work.

© 2025 Kramer A et al. *JAMA Network Open*.

eFigure 1. Example of a quarterly report sent to ICU Medical Directors.

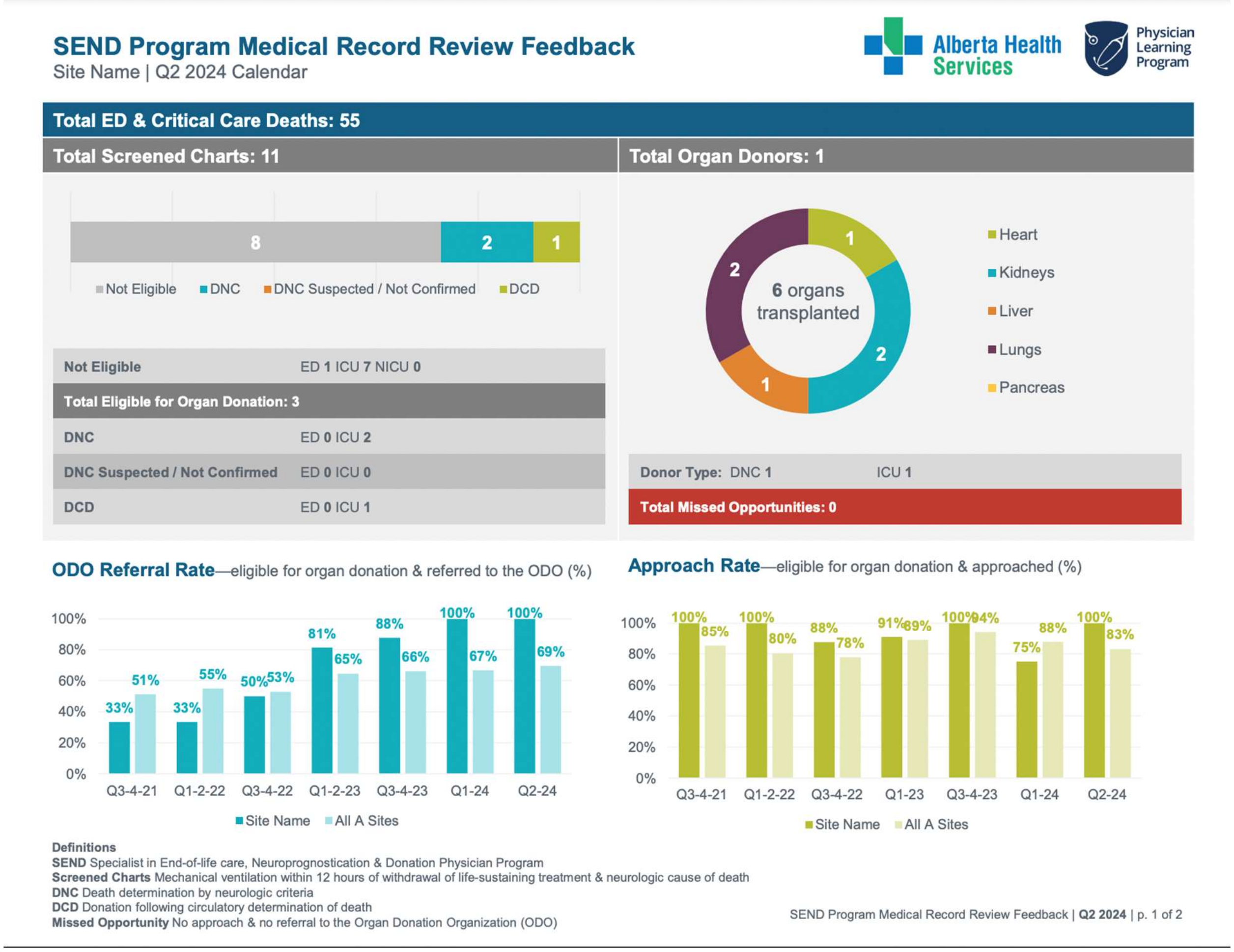

© 2025 Kramer A et al. *JAMA Network Open*.

Total Missed Donation Opportunities—no approach and no referral to the ODO

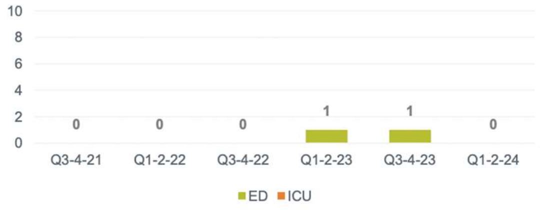

Historical Reference Data—total eligible, referred, approached, organ donors & organs transplanted

Type	2021 Q3-4	2022	2023	2024 Q1-2
Eligible	DNC **5**<br>DCD **4**	DNC **8**<br>DCD **3**	DNC **23**<br>DCD **11**	DNC **4**<br>DCD **3**
Referred	**3 / 9**	**5 / 11**	**27 / 34**	**7 / 7**
Approached	**9 / 9**	**10 / 11**	**32 / 34**	**6 / 7**
Donors	DNC **3**	DNC **2**	DNC **10**<br>DCD **3**	DNC **2**
Organs Transplanted	**16**	**4**	**38**	**10**

Comparable Site Determination—annual site classification criteria

Classification	Total patients eligible for organ donation in the prior year	Sites
**A**	**23 or more**	**FMC, PLC, RAH, UAH**
**B**	**7 to 22**	ACH, CRH, GNCH, MHRHC, STOL, SHC, RGH
**C**	**1 to 6**	GPRH, MIS, NLRHC, RDRH, SCH

Practice Recommendations

Mandatory Referral
Mandatory referral legislation came into place on April 1, 2023. Follow the Organ Donation Pathway to determine if a patient may be eligible for organ donation and whether a referral to Give Life Alberta is required. To reach the Donor Coordinator on call, use ROCA: Zone - Calgary – Area – Other – Service – Give Life Alberta – Donor Coordinator South (HOPE-SAOTDP) or via Switchboard @ 403-944-1212.

Registry Verification
25% of the adult population in Alberta has indicated their wishes for donation through the Alberta Organ and Tissue Donation Registry (AOTDR). The AOTDR was checked in **3** of the **3** patients (**100%**) who were eligible for organ donation in Q2. To determine if a patient has joined the registry, contact the Donor Coordinator on call.

Documentation
Of the charts in the SEND Medical Record Review **8** of **11 (77%)** included documentation of consideration of organ donation. Healthcare providers should document the outcome of donation consideration or donation conversations. For Connect Care, this is in the Discharge as Deceased Navigator.

Missed Donation Opportunities
A missed organ donation opportunity is a *never event*. Quality end-of-life care includes offering the option of organ donation to the families/substitute decision-makers of patients who may be eligible for organ donation

Resources
Organ Donation Pathway

Tissue Donation Pathway

Contact—for more information, please contact your local SEND physician

**Sample Site**	SEND MD Name & Contact Information

© 2025 Kramer A et al. *JAMA Network Open*.

eTable 1. Characteristics of eligible potential organ donors prior to initiation of a donation physician program based on whether death occurred prior to, or during the COVID-19 pandemic

	Pre-Pandemic (n=60) No.(%)	During Pandemic (n=69) No. (%)	P Value
Age (years, IQR)	52 (26-61)	51 (32-61)	0.88
Sex			
Male	40 (67)	49 (71)	0.59
Female	20 (33)	20 (29)	
Cause of Death			
HIBI	31 (52)	42 (61)	0.11
Cerebrovascular	12 (20)	18 (26)	
TBI	9 (15)	7 (10)	
Other	8 (13)	2 (3)	
Location			
ICU	60 (100)	65 (94)	0.06
ED	0	4 (6)	
Zone			
Edmonton	26 (43)	28 (41)	0.26
Calgary	30 (50)	30 (43)	
Regional	4 (7)	11 (16)	
Eligible Potential Donor Type			
DNC	33 (55)	36 (52)	0.75
DCC	27 (45)	33 (48)	
Missed Organ Donation Opportunity	19 (32)	24 (35)	0.71

Abbreviations: DCC=death by circulatory criteria; DNC=death by neurologic criteria; ED=emergency department; HIBI=hypoxic ischemic brain injury; ICU=intensive care unit; SEND=Specialist in end-of-life care, neuroprognostication, and donation; TBI=traumatic brain injury

© 2025 Kramer A et al. *JAMA Network Open*.

eFigure 2. Interrupted time series analysis comparing missed organ donation rate per month before and after mandatory referral legislation in Alberta.

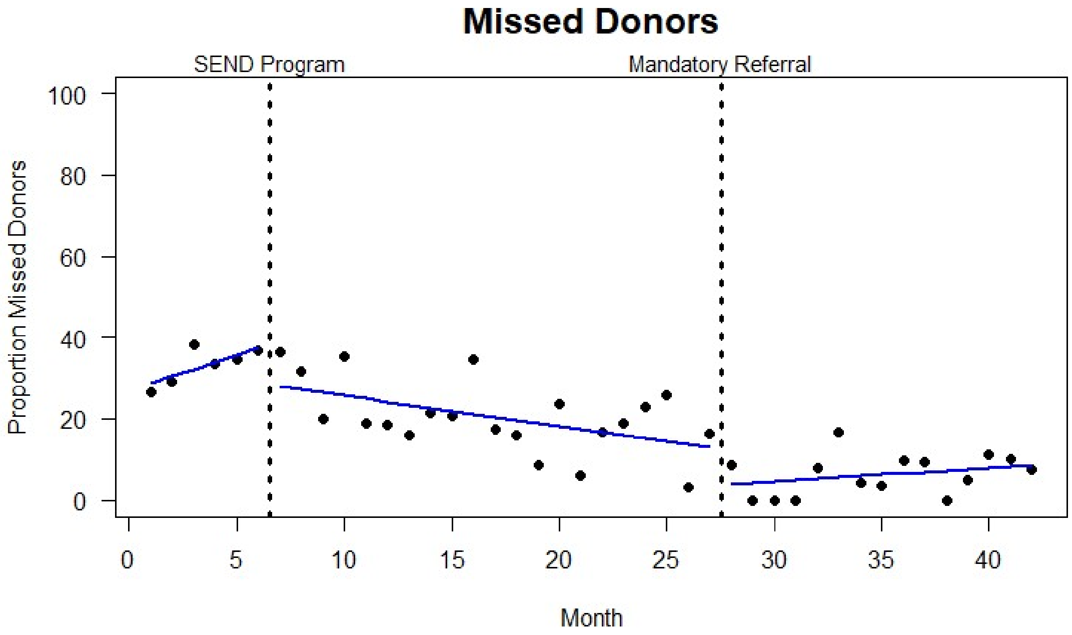

© 2025 Kramer A et al. *JAMA Network Open*.

eFigure 3. Interrupted time series analysis comparing referral of eligible potential organ donors, by month, before and after mandatory referral legislation in Alberta.

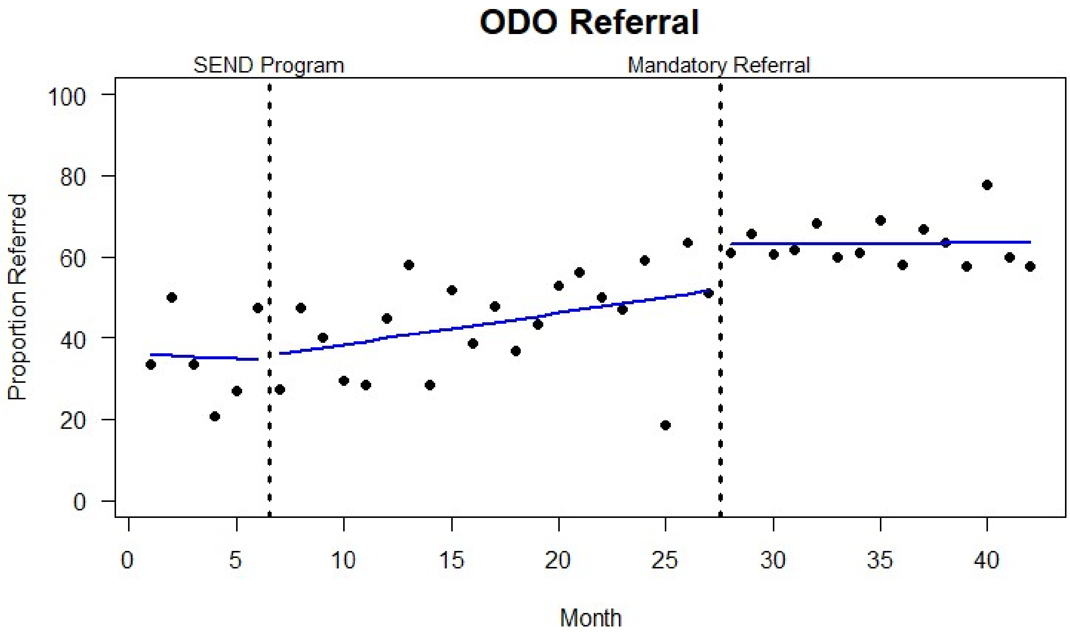

© 2025 Kramer A et al. *JAMA Network Open*.

eFigure 4. Interrupted time series analysis comparing the number of deceased organ donors per month before and after mandatory referral legislation in Alberta.

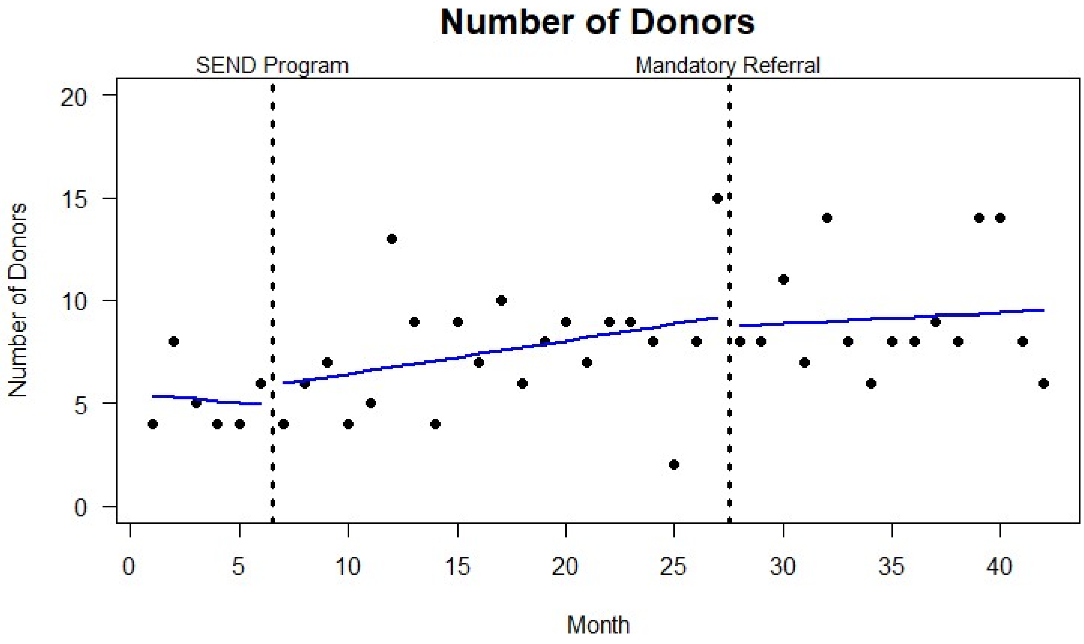

© 2025 Kramer A et al. *JAMA Network Open*.
