# Supplementary material for: Donation Physician Specialists and Missed Organ Donation Opportunities
Source: JAMA Netw Open. 2025 Aug 7;8(8):e2526067. doi: 10.1001/jamanetworkopen.2025.26067 (PMC12332632; doi:10.1001/jamanetworkopen.2025.26067)
Supplement: Supplement 2. — Data Sharing Statement [file jamanetwopen-e2526067-s002.pdf]

Data Sharing Statement

Kramer. Donation Physician Training and Missed Organ Donation Opportunities. *JAMA Netw Open*. Published August 07, 2025. doi:10.1001/jamanetworkopen.2025.26067

Data
Data available: No
